# Nanoparticle Enhanced Eutectic Reaction during Diffusion Brazing of Aluminium to Magnesium

**DOI:** 10.3390/nano9030370

**Published:** 2019-03-05

**Authors:** Tajwer S. Akhtar, Kavian O. Cooke, Tahir I. Khan, Mohammad Ali Shar

**Affiliations:** 1 School of Engineering and Informatics, University of Bradford, Bradford BD7 1DP, West Yorkshire, UK; tajwer_akhtar@hotmail.com (T.S.A.); t.khan20@bradford.ac.uk (T.I.K.); M.Baloch@bradford.ac.uk (M.A.S.); 2 Mechanical Engineering Department, University of Technology, Jamaica, 237 Old Hope Road, Kingston, Jamaica; 3 King Abdullah Institute for Nanotechnology, King Saud University, Riyadh 11451, Saudi Arabia

**Keywords:** microstructure, transient liquid phase diffusion brazing, nanoparticles, interlayer, electrodeposited

## Abstract

Diffusion brazing has gained much popularity as a technique capable of joining dissimilar lightweight metal alloys and has the potential for a wide range of applications in aerospace and transportation industries, where microstructural changes that will determine the mechanical and chemical properties of the final joint must be controlled. This study explores the effect of Al_2_O_3_ nanoparticles on the mechanical and microstructural properties of diffusion brazed magnesium (AZ31) and aluminium (Al-1100) joints. The results showed that the addition of Al_2_O_3_ nanoparticle to the electrodeposited Cu coating increased the volume of eutectic liquid formed at the interface which caused a change to the bonding mechanism and accelerated the bonding process. When the Cu/Al_2_O_3_ nanocomposite coatings were used as the interlayer, a maximum bond strength of 46 MPa was achieved after 2 min bonding time while samples bonded using pure-Cu interlayers achieved maximum strength after 10 min bonding time. Chemical analysis of the bond region confirmed that when short bonding times are used, the intermetallic compounds formed at the interface are limited to the compounds consumed in the eutectic reaction.

## 1. Introduction

Interest in lightweight materials has significantly increased in recent years, driven by demands for fuel consumption and weight reduction in the transportation and aerospace industries. As the lightest structural metal available, magnesium alloys could provide greater benefits over metals like aluminium and steel to further reduce costs associated with fuel consumption and weight reduction. The use of magnesium alloys requires the ability to join these materials to other structural materials such as steel and aluminium [1]. The strength requirements of some automobile components requires bi-metallic joining is an essential criterion for optimizing the design of the component during manufacturing [2].

The challenges encountered in bi-metallic joining include differences in the thermo-kinetic and mechanical properties of the material such as: crystal structure, melting point, and thermal conductivity. These limitations often result in the formation of intermetallic compounds that weakens the joints [1]. Technologies capable of bi-metallic joining include tungsten inert gas welding (TIG) [3], metal inert gas welding (MIG) [4], and resistance spot welding (RSW) [5,6]. The heat input used in arc welding processes leads to the formation of a large heat affected zone (HAZ) and fusion zones (FZ) with properties that are significantly different from those of the parent materials. Additionally, the presence of tenacious surface oxides can lead to the formation of porosities or material distortion due to the difference in the melting temperature of the metal and oxides. Laser welding [7] and ultrasonic spot welding (USSW) [8] have also shown the potential for bi-metallic joining of aluminium and magnesium alloys. When suitable interlayers are used between the two materials during LW and USSW the volume of intermetallic compounds formed within the weld also decreases.

Solid-state welding processes such as Friction Stir Welding (FSW) and diffusion bonding have been shown to have the potential to join Mg alloys without intermetallic formation. Numerous studies have indicated that FSW of bi-metallic joints containing magnesium and aluminium reduces the volume of intermetallic compounds that form during the welding process, however, cracking within the weld is a common defect encountered [9,10,11]. Diffusion bonding, however, shows great potential for preventing intermetallic formation during bonding since the bonding process is performed at low temperatures. During the diffusion brazing process, a thin liquid layer is formed at the interface either due to direct melting of the interlayer or by eutectic reaction between the interlayer and the base metals removes surface oxides and wets the surface of the base metals [12]. Several studies have explored dissimilar joining of Mg alloys. Some of the interlayers that have been studied include Ni [13], Sn, Cu [14], Ni/Al_2_O_3_ [15], and Zn. The results of these studies have shown that diffusion brazing is capable of joining magnesium alloys to other metals while reducing the volume of intermetallic compounds formed during the bonding process.

In this study, bi-metallic diffusion brazing of magnesium (AZ31) and aluminium (Al1100) alloys were investigated to determine the effect of dispersed Al_2_O_3_ nanoparticles on the mechanical and microstructural properties of the joints formed. Differential thermogravimetric analysis was performed to evaluate the impact of the nanoparticles on the eutectic reaction during bonding. Finally, a comparative analysis is presented of the properties developed in joints made using pure-Cu and Cu/Al_2_O_3_ nanocomposite coatings.

## 2. Materials and Methods

Commercially available magnesium (AZ31) and aluminium (Al-1100) alloys were used in this study. The chemical composition of each material is summarized in Table 1.

The test materials were cut to dimensions of 10 mm × 10 mm × 10 mm using a Struers Accutom-5 cutting machine equipped with a diamond cut-off wheel. The bonding surfaces were prepared by grinding with abrasive papers from 240 to 2500 grit and polished to 1-micron using particle impregnated polishing compound and degreased with acetone. Each sample was cleaned with a mixture of distilled water and acetone in an ultrasonic bath for 10 s.

The bonding process was carried out in a bespoke diffusion bonding machine equipped with a Flexitune induction heater (Flexitune 9, Inductotherm Heating and Welding Hampshire, UK). Samples to be joined were assembled at room temperature and placed on mounting stage within the induction coil. The bonding temperature was monitored by an ungrounded K-type thermocouple was inserted into a 1 mm diameter hole drilled 2 mm from the faying surfaces. A uniaxial pressure of 0.2 MPa was applied to the sample to ensure good contact between the faying surfaces. When a vacuum of 2.0 × 10^−2^ torr was achieved, the samples were heated to 500 °C and held at the bonding temperature for the prescribed duration. Two types of interlayers were studied; pure-Cu and Cu/Al_2_O_3_ electrodeposited coatings. Bonding times of 2, 5, and 10 min were used. At the end of the bonding process, the equipment was turned off and the sample cooled to room temperature in a vacuum. After bonding, the samples were cut transverse through the bond-line using Struers Accutom-5 cutting machine (Struers ApS, Ballerup, Denmark) and mounted in Bakelite. After mounting, the samples were grinded using abrasive papers progressively from 240 to 1200 grit and polished using 1 µm particle impregnated carrier paste and etched with a drop of 1% hydrofluoric acid (HF) (BDH Chemicals, Poole, UK) in acetone for 2 s.

The mechanical properties of the joints were evaluated using Vicker’s micro-hardness testing and shear strength measurements. Vickers micro-hardness measurements were performed on the cross-section of the bonded samples using a 7640 Leitz Wetzlar hardness tester (Ernst Leitz Ltd. Midland, ON, Canada) with a load of 50 g and a dwell time of 30 s. Each indentation was made at 100 µm spacing and average over five data points. A single lap shear test was used to evaluate the strength of the bonds formed. Microscopic and chemical analyses of the bonded samples were performed using a scanning electron microscope (SEM) equipped with energy dispersive spectroscopy (EDS) (FEI 400, Oxford, UK). XRD analysis was performed using an X′ pert analytical diffractometer (Malvern Panalytical Ltd, Malvern, UK) with the following settings; 40 kV, 40 mA, step size 0.1, 2-Theta ranging from 10° to 90° and measured at 1 s per step. The thermal measurements were carried out on a TA instruments SDT-Q-600 thermal analysis system (TA Instruments Ltd, New Castle, UK) in nitrogen atmosphere with a heating rate of 10 °C/min for a temperature range of 20–540 °C.

### Electrodeposition Process

**Figure 1:**
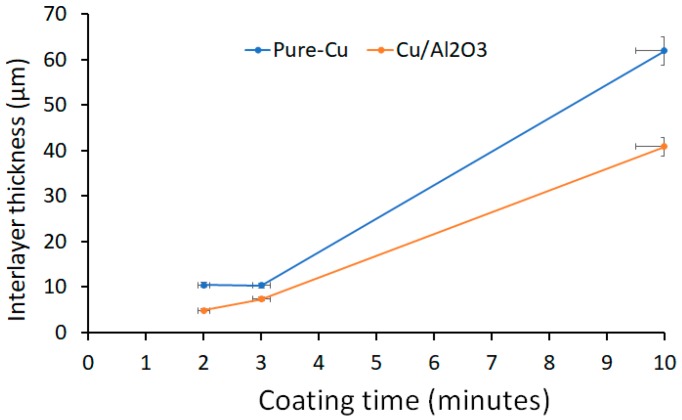
The relationship between the coating time and the thickness of the deposited coatings studied.

Prior to bonding, the aluminium sample was electroplated with Cu containing Al_2_O_3_ nanoparticles. Electrodeposition was carried out in a 250 mL beaker using a sulphate bath with the following composition: 200 g/L copper sulphate (CuSO_4_·H_2_O) (Fisher Scientific UK Ltd, Loughborough, UK), 50 mL/L Sulphuric Acid (BDH Chemicals, Poole, UK) and 1 litre of distilled water. Bath-1 was used to deposit pure Cu coatings, while 20 g/L of Al_2_O_3_ powder with a particle size of 40 nm was added to bath-2 for the deposition of nanostructured Cu/Al_2_O_3_ coatings. Each coating was prepared at room temperature using a current density of 3 A/dm^2^, and stir rate 200 rpm. A coating thickness of 20 µm was deposited using the relationship shown in Figure 1. EDS analysis of the Cu/Al_2_O_3_ coating found approximately (8.43 wt%) of Al_2_O_3_ particles were uniformly distributed through the thickness of the coating.

## 3. Results and Discussion

### 3.1. Bond Formation—Interfacial Contact and Solid-State Diffusion

**Figure 2:**
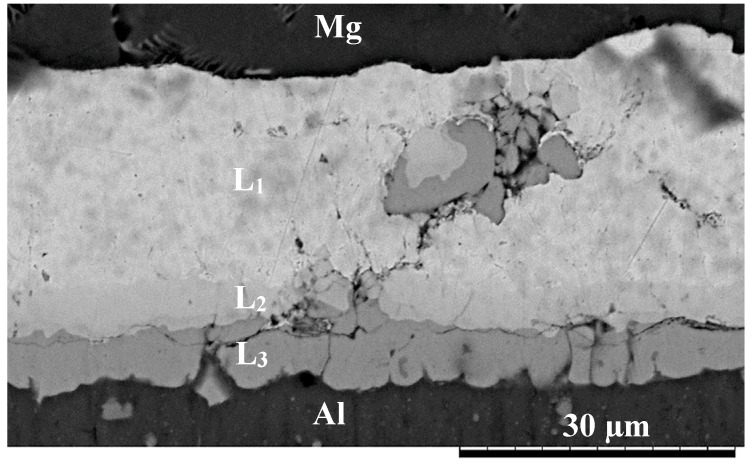
Scanning electron microscope (SEM) micrograph of the joint made using 20 µm Cu/Al_2_O_3_ and bonded at 500 °C for 1 min.

During the first stage of the bonding process, interfacial contact was achieved due to the application of an external static load which presses that sample together during the heating stage. Deformation of the surface asperities during the heating stage ensures intimate contact of the bonding surfaces. As the temperature of the samples increased, interdiffusion at the Mg/interlayer and Al/interlayer interface results in the formation of three reaction layers as shown in Figure 2. EDS analysis revealed that L_1_ is Cu containing dispersed Al_2_O_3_ nanoparticles. L_2_, on the other hand, appears to be a copper aluminide intermetallic with composition 34.78 (wt%) Al and 41.68 (wt%) Cu while L_3_ had a composition 42.19 (wt%) Cu and 56.68 (wt%) Al. According to the Al–Cu phase diagram, the L_3_ phase is likely to be Cu_2_Al intermetallic compound [16].

### 3.2. Liquid Formation and Base Metal Dissolution

**Figure 3:**
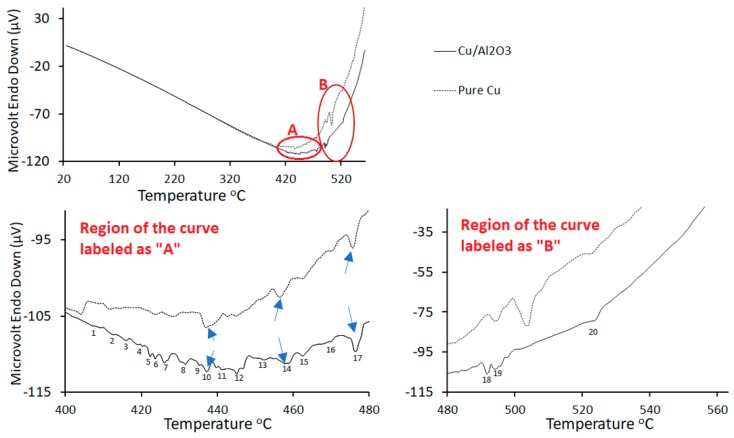
Thermogravimetric analytical curves of the Mg/Cu/Al and Al/Cu-Al_2_O_3_/Mg joints. The analysis was performed in nitrogen having a flow rate of 40 mL/min and a heating rate of 10 °C/min.

The effect of Al_2_O_3_ nanoparticles on the formation of the liquid phase during the bonding process was studied using differential thermogravimetric analysis. The results of the thermogravimetric analyses are presented in Figure 3 and show significant differences between the number of endothermic peaks formed when the pure-Cu interlayer was used when compared to the number of endothermic peaks formed when Cu/Al_2_O_3_ nanocomposite interlayer was used. In both analyses, matching peaks were labelled as 10, 14, and 17. Figure 3 shows the formation of several other endothermic reactions when Cu/Al_2_O_3_ interlayer was used. The presence of these peaks suggests the occurrence of other liquid forming reactions within the samples and at the interface during bonding. The peaks labelled as 18 and 19 for the Cu/Al_2_O_3_ interlayer appear to occur at a lower temperature than the matching peaks for the pure-Cu interlayer. The number of small endothermic reactions formed when the Cu/Al_2_O_3_ interlayer are believed to be responsible to the larger volume of eutectic liquid formed at the joint interface. Table 2 is a list of possible liquid forming reactions in the Al-Cu-Mg system with melting temperatures matching the endothermic peaks seen when the CuAl_2_O_3_ interlayer was used are presented in Table 2 [17]. Holding of the samples at the bonding temperature, resulted in the spreading of the liquid between the bonding surfaces under the effect of the applied pressure and capillary action. As the contact area between the surfaces increased, interdiffusion between Al, Cu, and Mg also increased.

**Figure 4:**
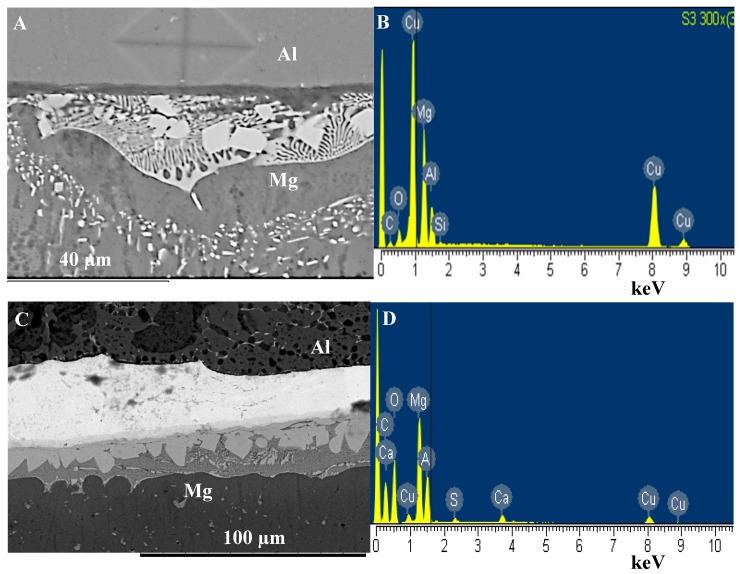
Eutectic microstructure formed at the joint interface during diffusion brazing. (**A**) Eutectic microstructure formed using pure-Cu. (**B**) Energy dispersive spectroscopy (EDS) spectrum of region-1. (**C**) Eutectic microstructure formed using Cu/Al_2_O_3_ interlayer. (**D**) EDS spectrum of region-2.

**Figure 5:**
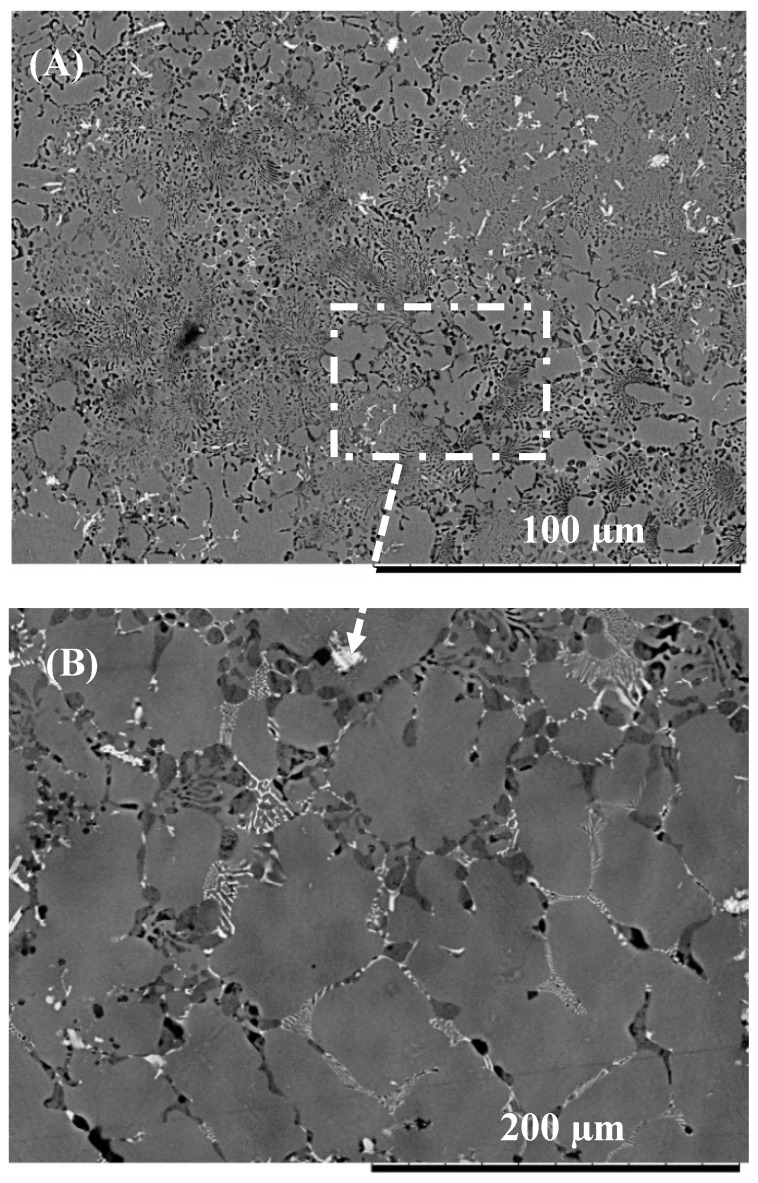
(**A**) Eutectic liquid formation within the Mg-side of the sample (**B**) Detailed view of the highlighted region showing eutectic liquid along the grain boundary.

Microstructural analysis of the joint zone (see Figure 4A) shows the presence of a eutectic microstructure at the Mg-side of the interface which confirms the formation of the eutectic reaction during bonding. When Cu/Al_2_O_3_ as used as the interlayer a larger eutectic microstructure was observed at the interface as shown in Figure 4C. Extensive melting was also observed at the grain boundaries within the Mg sample as shown in Figure 5 when both pure-Cu and Cu/Al_2_O_3_ interlayer were used.

### 3.3. Isothermal Solidification

**Figure 6:**
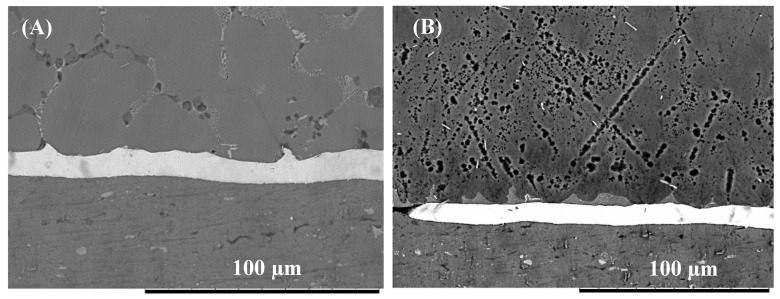
(**A**) SEM micrograph of the Mg/Al bonded for 2 min using Cu interlayer. (**B**) Mg/Al bonded for 5 min using pure-Cu interlayer.

**Figure 7:**
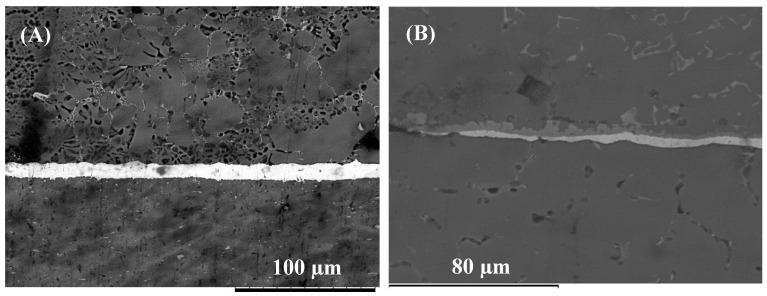
(**A**) SEM micrograph of Mg/Al joint bonded for 10 min using pure-Cu interlayer (**B**) Detail view of the interface.

The effect of bonding time on joint microstructure was studied and compared as a function of interlayer composition. Figure 6 presents the microstructure of the joint bonded using pure-Cu interlayer for 2 min. Analysis of the joint showed that after two min, a thick Cu interlayer remained at the interface. Evidence of eutectic microstructures was observed at the grain boundaries within the Mg bulk material. When the bonding time was increased to 5 min, the image shows evidence of the eutectic melting along the Cu/Mg interface, which is believed to have occurred due to the eutectic reaction as shown as peak 17 (Table 2). Sections of the interface show that the Cu interlayer had completely diffused into the base metals. Increasing the bonding time to 10 min enhanced the interdiffusion between the Cu interlayer and the Al and Mg base metals. The change in the composition of the joint zone led to the formation of a blocky reaction layer at the Cu/Al interface as shown in Figure 7. EDS analysis of the blocky structures suggests the formation of the compound CuAl_2_ with a composition of 32.5 wt% Cu and 64.3 wt% Al.

**Figure 8:**
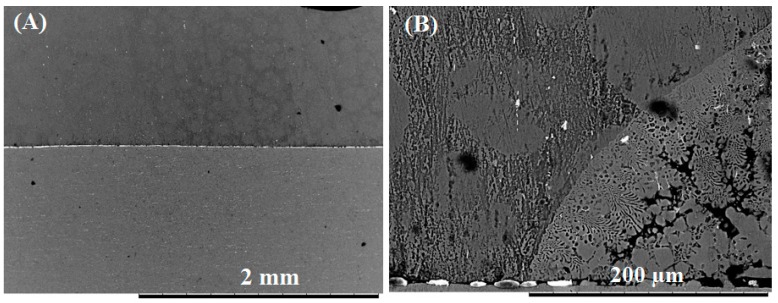
(**A**) SEM micrograph of Mg/Al bonded for 2 min using Cu /Al_2_O_3_ interlayer: (**B**) Detail view of the interface.

**Figure 9:**
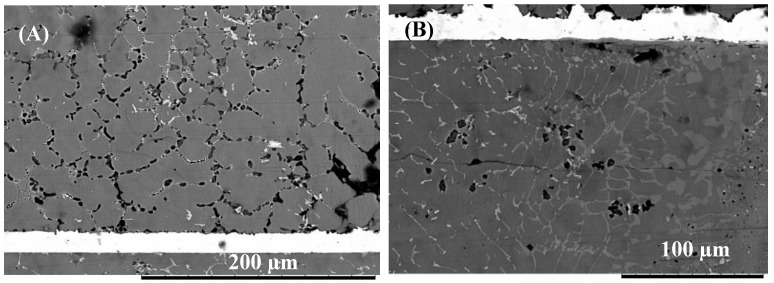
(**A**) SEM micrograph of Mg/Al bonded for 5 min using Cu/Al_2_O_3_ interlayer. (**B**) Bond interface.

**Figure 10:**
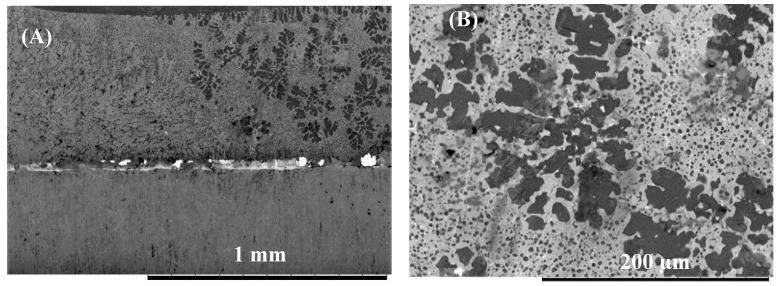
(**A**) SEM micrograph of Mg/Al bonded for 10 min using Cu/Al_2_O_3_ interlayer. (**B**) Detailed view of the interface.

When the Cu/Al_2_O_3_ coating was used as the interlayer, significant differences were observed within the joint zone as shown in Figure 8. For a bonding time of 2 min, a large volume of the eutectic microstructure was observed at the Mg/interlayer interface. The results also showed that the Cu/Al_2_O_3_ interlayer was not completely dissolved in the eutectic reaction. However, the presence of the Al_2_O_3_ nanoparticles is believed to be responsible for the small grains observed within the joint zone when compared to grain size observed when pure-Cu was used as the interlayer. When the bonding time was increased to 5 min two reaction layers were observed at the Al/interlayer interface (see Figure 9). EDS analysis revealed that the reaction layers had a composition of 33.2 wt% Cu and 66.3 wt% Al which is indicative of CuAl_2_ intermetallic compounds. Further increase of the bonding time to 10 min resulted in the most significant microstructural changes within the joint zone as shown in Figure 10. The Mg-side of the joint exhibited significant grain growth and the formation of dark grey blocky leaf-like structures within, adjacent to the bond-line. EDS analysis of the joint suggests that the interdiffusion of Cu and Mg lead to the formation of CuMg_2_ intermetallic compound within the joint affected zone as shown in Figure 10B. The softening of the Mg due to eutectic melting along the grain boundaries is believed to have increased the rate of diffusion and accelerated the homogenization phase of the bonding process. The higher rate of diffusion of Mg into Cu (2 × 10^−13^ cm/s) than Cu into Mg (5.8 × 10^−14^ cm/s), caused intermetallic compound CuMg_2_ to form within the joint affected region adjacent to the bond-line.

## 4. Mechanical Performance of the Bonds

### 4.1. Micro-Hardness Measurements

**Figure 11:**
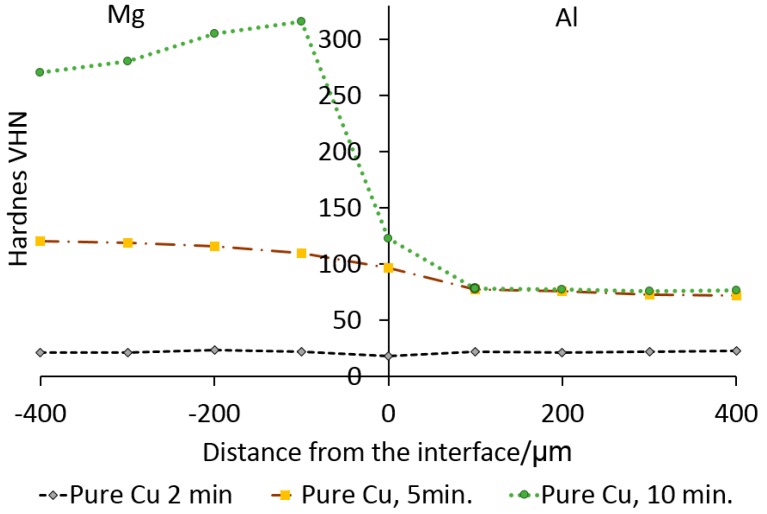
Micro-hardness profile for Mg/Al bonds as a function of bonding time using pure Cu interlayer.

Figure 11 shows the results obtained from the micro-hardness test of Mg/Al joint using pure-Cu interlayer. The results show that the hardness within the joint zone (JZ) increase with increasing bonding time. The hardness on the Mg-side of the JZ is higher than the hardness of the Al-side of the joint with peak hardness of 316 VHN occurring at 100 µm from the interface and decreasing to 79 HV at the Al side. The peak hardness of 122 VHN was recorded at the interface for a bonding time of 10 min.

**Figure 12:**
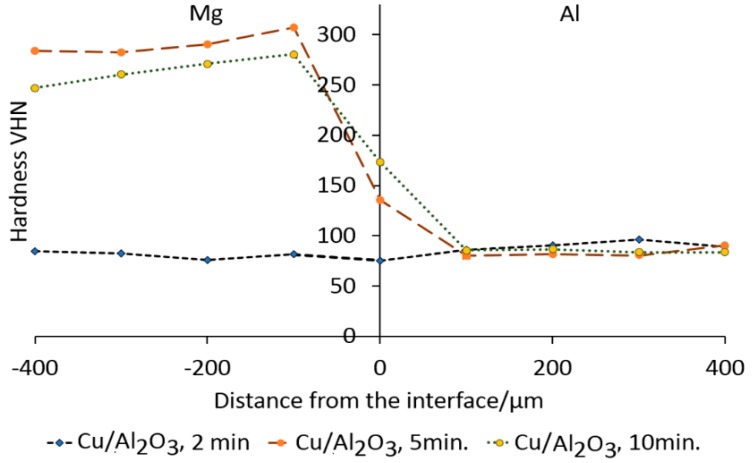
Micro-hardness profile for Mg/Al bonds as a function of bonding time using Cu/Al_2_O_3_ interlayer.

The micro-hardness values taken across joints made using the Cu/Al_2_O_3_ interlayer are presented in Figure 12. The results show that the average hardness for 2 min bonding time fluctuated around 79 VHN which was higher than the values recorded for joints made with pure-Cu interlayer. When the bonding time was increased, a corresponding increase in hardness was recorded in the JZ. The peak hardness of 305 VHN was recorded at 100 µm from the interface and decreased to 79 HV at the Al side. Further increase of the bonding time to 10 min caused a reduction of the peak hardness value. The highest hardness value at the interface appeared to be 173 VHN at 10 min and the lowest value of 84 VHN was observed at 5 min.

The results of the hardness testing confirm that the composition of the interlayer has a significant effect on the hardness variation across the JZ. The results showed a difference in the hardness values for samples bonded for 2 min with pure Cu and Cu/Al_2_O_3_ interlayers. The higher hardness recorded when the Cu/Al_2_O_3_ interlayer was used is attributed to the presence of the Al_2_O_3_ nanoparticles in the interlayer. The increase in hardness on the Mg-side of the JZ was attributed to the migration of the Al_2_O_3_ nanoparticles during the melting and widening stages of the bonding process, and the formation of CuMg_2_ intermetallic compounds within the joint region. This type of dispersion strengthening of the joint region was predicted by the Zener equation [18].

### 4.2. Shear Strength Measurements

**Figure 13:**
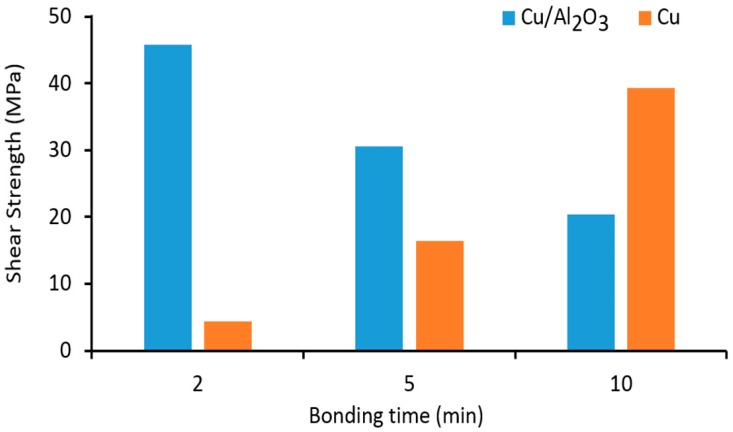
Shear strength measurements as a function of bonding time using Cu/Al_2_O_3_ and pure-Cu interlayers.

A comparison of the shear strength measurements presented in Figure 13 revealed that for the samples bonded with a Cu/Al_2_O_3_ interlayer gave a maximum bond strength of 45.8 MPa when bonding time of 2 min was used. This represented 67.5 percent of the shear strength of the aluminium 1100 base metal. When the bond time was increased to 5 min, the bond strength decreased to 30.6 MPa. Further increase in the bond time to 10 min similarly caused a reduction of the bond strength to 20.4 MPa. When compared to samples bonded with pure-Cu coatings, the opposite relationship was observed between bond time and bond strength. For samples bonded for 2 min using pure Cu, interlayer bond strength of 4.4 MPa was achieved. When the bond time was increased to 5 min the bond strength also increased to 16.4 MPa. The maximum bond strength occurred for a bonding time of 10 min with pure-Cu interlayer. An inverse proportional relationship was observed between bond strength and bonding time for samples bonded using Cu/Al_2_O_3_ interlayers while a more directly proportional relationship was observed when pure-Cu interlayer was used, i.e., the bond strength increased with increasing bonding time.

The significant differences observed in the strength development of the two types of interlayers was attributed to the presence of two distinct bonding mechanisms. For the Cu/Al_2_O_3_ interlayer the bonding mechanism was characteristic of brazing during which the eutectic liquid wets the faying surfaces and spreads by capillary action and the applied shear force. On the other hand, when the pure-Cu interlayer was used, a smaller volume of eutectic liquid formed at the interface. As such, the bond strength developed through continued interdiffusion between the interlayer and the base metals thus causing a change of the composition within the bond region and formation of various copper aluminides which strengthens the bond.

**Figure 14:**
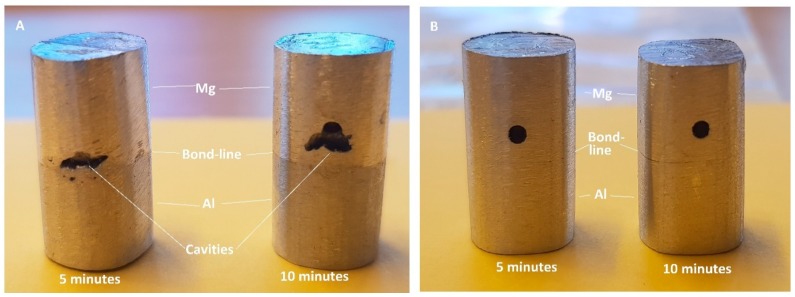
Bonded samples used for shear testing: (**A**) Sample bonded with Cu/Al_2_O_3_ interlayer. (**B**) Samples bonded using pure-Cu interlayer.

The difference in the volume of eutectic liquid formed during bonding was also evident on the shear stress samples shown in Figure 14. When the shear force was applied during bonding, some of the eutectic liquid squeezed out when the Cu/Al_2_O_3_ interlayer was used, leaving cavities in the joint affected regions as shown in Figure 14A. When compared to samples bonded using pure-Cu interlayer (see Figure 14B), liquid squeeze-out was prevented, since a smaller volume of liquid was formed. If the thickness of the Cu/Al_2_O_3_ interlayer is reduced, the volume of liquid formed during bonding can be reduced which would prevent cavities from forming during bonding [19]. Additionally, since large external pressure is not required for diffusion brazing, a lower applied load during bonding could also prevent expulsion of the eutectic liquid.

**Figure 15:**
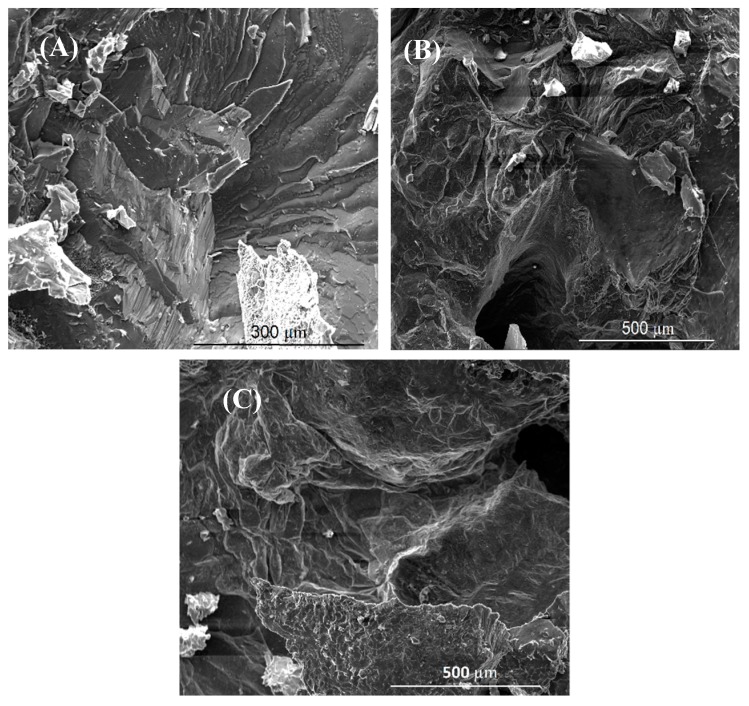
SEM images of the fracture surfaces for joints bonded with Cu/Al_2_O_3_ for (**A**) 2 min, (**B**) 5 min and (**C**) 10 min.

**Figure 16:**
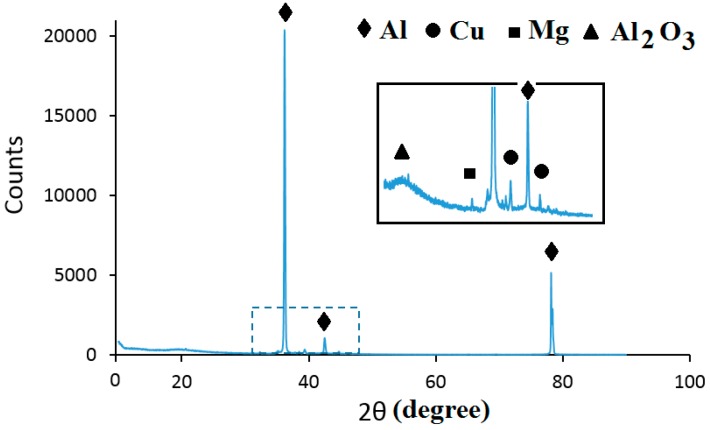
XRD spectrum of the fracture surface for joints bonded with Cu/Al_2_O_3_ for 2 min.

The fracture surface of a bond made using Cu/Al_2_O_3_ interlayer for 2 min is shown in Figure 15. The fracture surface was characterized by an acicular structure with angular cleavage planes typical of transgranular fracture. The undulating nature of the surface and the large ridges formed suggest that the fracture propagated through the joint region adjacent to the bond-line at the Al interface. The presence of small grains at the interface is believed to be responsible for the high strength recorded. The XRD analysis of the fracture surface presented in Figure 16 confirmed that intermetallic compounds were not formed when a bonding time of 2 min was used.

When the bonding time was increased to 5 min, the fracture surface presented in Figure 15B showed the presence of large cleavage planes suggesting brittle intergranular fracture along the bond-line. The increased bonding time facilitated the formation of intermetallic compounds within the joint zone. Similar findings were observed when the bonding time was increased to 10 min as shown in Figure 15C. The presence of cleavage planes at the fracture surfaces and the increase in the size of the cleavage planes suggested that the brittleness of the joint increased with bonding time. This finding was also confirmed by the hardness test data which showed that the hardness within the joint zone increased with increasing bonding time.

**Figure 17:**
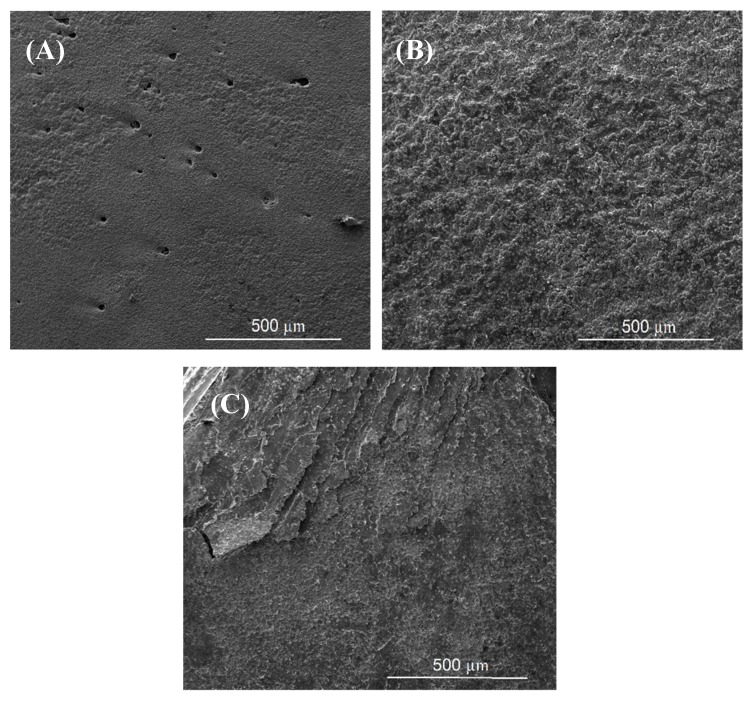
SEM images of the fracture surfaces for joints bonded with pure-Cu for (**A**) 2 min, (**B**) 5 min and (**C**) 10 min.

**Figure 18:**
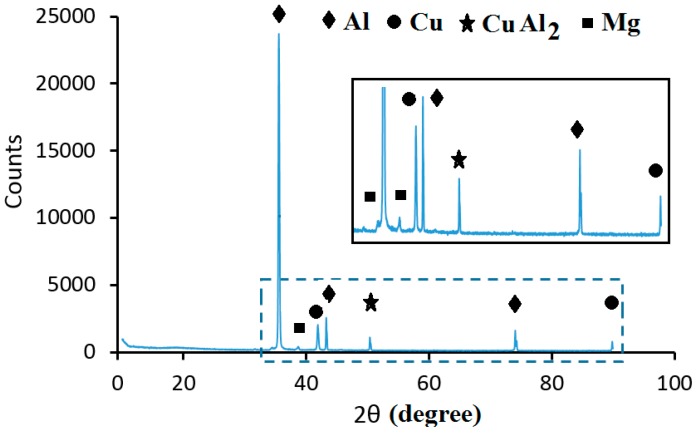
XRD spectrum of the fracture surface for joints bonded with pure-Cu for 10 min.

Figure 17 shows the fracture surface of the sample bonded for 2 min using pure-Cu as the interlayer. Analysis of the fracture surface revealed the presence of small dimples on the surface where bonding had taken place. When the bonding time was increased to 5 min the fracture surface showed the presence of a rough undulating surface with shallow depressions indicative of a pull-out fracture. Further increase of the bonding time to 10 min led to the formation of numerous cleavage planes and delaminated regions of the fracture surface. Cracks propagated through the bond-line at the Al/Cu interface. XRD analysis of the sample bonded for 10 min is presented in Figure 18 and revealed a small peak for the intermetallic compound CuAl_2_.

## 5. Conclusions

Transient liquid phase diffusion brazing of Al-1100 to Mg-AZ31 was successfully achieved using a Cu/Al_2_O_3_ interlayer. The compositional variation across the joint zone was assessed using EDS analysis and Vickers microhardness testing. The results showed that the average hardness within the joint zone increased with increasing bonding time for the joints formed with both Cu/Al_2_O_3_ and pure-Cu interlayers. The EDS data confirmed that the changes observed in the hardness values were caused by intermetallic compounds forming within the joint zone at the Mg interface.

Thermogravimetric analysis of the bonding couples; Al/Cu/Mg and Al/Cu-Al_2_O_3_/Mg showed that the addition of Al_2_O_3_ nanoparticles to the copper coating had a twofold effect of on the eutectic melting at the interface. Firstly, the volume of liquid that formed at the interface increased when compared to the volume formed when pure-Cu was used. Secondly, the particles served as a melting point depressant causing the eutectic temperature to decrease.

Shear strength analyses indicated that a maximum joint strength of 46 MPa was achieved after a bonding time of 2 min when Cu/Al_2_O_3_ was used as the interlayer. Further increase of the bonding time resulted in a reduction of the joint strength. On the other hand, when pure-Cu was used as the interlayer, the joint strength increased consistently with increasing bonding time from 4.4 MPa after 2 min to 38 MPa after 10 min. EDS and XRD analyses of the joint regions and the fracture surfaces showed that the volume of CuAl_2_ intermetallic compound increased with increasing bonding time.

Two distinct mechanisms of bonding were observed for interlayers studied. For the Cu/Al_2_O_3_ interlayer, the bonding mechanism was more characteristic of brazing during which the eutectic liquid wets the faying surfaces and spreads by capillary action and the applied shear force. On the other hand, when the pure-Cu interlayer was used, a smaller volume of eutectic liquid formed at the interface. As such, bond strength develops through continued interdiffusion between the interlayer and the base metals, which changes the composition of the bond region due to the formation of various copper aluminides which strengthen the bond.

## Figures and Tables

**Table 1:** Chemical composition (wt%) of materials studied.

Materials	Mg	Al	Si	Zn	Mn
Mg (AZ31)	Bal.	2.66	0.03	1.01	0.37
Al1100	-	Bal.	0.9	0.1	0.05

**Table 2:** Liquid forming reactions occurring at the interface and within the grain boundary of the Mg-side during the bonding process.

Peak Number	Reaction		Composition (at%)		
		Phase	Al	Cu	Mg
1	${\mathrm{Mg}}_{2}{\mathrm{Al}}_{3}+{\mathrm{Mg}}_{17}{\mathrm{Al}}_{12}\;\overset{{409.8\;}^{{}^{\circ}}C}{\to }{\mathrm{Mg}}_{23}{\mathrm{Al}}_{30},\;T$	T	55.2	3.4	41.4
5	$L\;\overset{{424.7\;}^{{}^{\circ}}C}{\to }\mathrm{Mg}+T+{\mathrm{Mg}}_{17}{\mathrm{Al}}_{12}$	T	47.9	9.2	42.9
7	$L+Q\overset{{426.8\;}^{{}^{\circ}}C}{\to }T+\left(\mathrm{Mg}\right)$	T Q	47.8 43.8	9.3 18.7	42.9 37.5
12	$L\;\overset{{447.6\;}^{{}^{\circ}}C}{\to }T+{\mathrm{Mg}}_{2}{\mathrm{Al}}_{3}+{\mathrm{Mg}}_{17}{\mathrm{Al}}_{12}$	T	55.4	4.1	40.5
13	$L\;\overset{{447.6\;}^{{}^{\circ}}C}{\to }T+{\mathrm{Mg}}_{2}{\mathrm{Al}}_{3}+\left(\mathrm{Al}\right)$	T	55.1	3.4	41.5
14	$L\;+\lambda_{2}\overset{{454.6\;}^{{}^{\circ}}C}{\to }Q+\left(\mathrm{Mg}\right)$	Q $\lambda_{2}$	43.8 37.2	18.7 29.4	37.5 33.4
15	$L\;+S\overset{{469.2\;}^{{}^{\circ}}C}{\to }T+\left(\mathrm{Al}\right)$	T S	52.4 50	8.1 25	39.4 25
16	$L\;+Q\overset{{479\;}^{{}^{\circ}}C}{\to }T+S$	T S	52 50	8.3 25	39.7 25
17	$L\;\overset{{476\;}^{{}^{\circ}}C}{\to }\left(85.5\%\right)\mathrm{Mg}+\left(14.5\%\right){\;\mathrm{Mg}}_{2}\mathrm{Cu}$	-	-	-	-
18	$L\;\overset{{481.2\;}^{{}^{\circ}}C}{\to }\lambda_{1}+{\mathrm{CuMg}}_{2}+\mathrm{Mg}$	$\lambda_{1}$	19.2	47.1	33.7
19	$L\;+\lambda_{1}\overset{{497.3\;}^{{}^{\circ}}C}{\to }\lambda_{2}+\mathrm{Mg}$	$\lambda_{1}$	34.6	32	33.4
		$\lambda_{2}$	31.0	35.5	33.5
22	$L\;\overset{{546\;}^{{}^{\circ}}C}{\to }\mathrm{Al}+{\;\mathrm{CuAl}}_{2}$	-	-	-	-
